# Severe fever with thrombocytopenia syndrome with central nervous system symptom onset: a case report and literature review

**DOI:** 10.1186/s12883-024-03664-6

**Published:** 2024-05-10

**Authors:** Dawei Shan, Weibi Chen, Gang Liu, Huimin Zhang, Shuting Chai, Yan Zhang

**Affiliations:** Department of Neurology, Xuanwu Hospital, Capital Medical University, Beijing, 100053 China

**Keywords:** Severe fever with thrombocytopenia syndrome, Novel bunyaviruses, Central nervous system, Encephalitis, Involuntary shaking

## Abstract

**Background:**

Severe fever with thrombocytopenia syndrome (SFTS) is a natural focal disease transmitted mainly by tick bites, and the causative agent is SFTS virus (SFTSV). SFTS can rapidly progress to severe disease, with multiple-organ failure (MOF) manifestations such as shock, respiratory failure, disseminated intravascular coagulation (DIC) and death, but cases of SFTS patients with central nervous system (CNS) symptoms onset and marked persistent involuntary shaking of the perioral area and limbs have rarely been reported.

**Case presentation:**

A 69-year-old woman with fever and persistent involuntary shaking of the perioral area and limbs was diagnosed with SFTS with CNS symptom onset after metagenomic next-generation sequencing (mNGS) of cerebrospinal fluid (CSF) and peripheral blood identified SFTSV. The patient developed a cytokine storm and MOF during the course of the disease, and after aggressive antiviral, glucocorticoid, and gamma globulin treatments, her clinical symptoms improved, her laboratory indices returned to normal, and she had a good prognosis.

**Conclusion:**

This case gives us great insight that when patients with CNS symptoms similar to those of viral encephalitis combined with thrombocytopenia and leukopenia are encountered in the clinic, it is necessary to consider the possibility of SFTS involving the CNS. Testing for SFTSV nucleic acid in CSF and blood (mNGS or polymerase chain reaction (PCR)) should be carried out, especially in critically ill patients, and treatment should be given accordingly.

**Supplementary Information:**

The online version contains supplementary material available at 10.1186/s12883-024-03664-6.

## Background

Severe fever with thrombocytopenia syndrome (SFTS) is a natural focal disease transmitted mainly by tick bites, and the causative agent is a novel Bunyavirus, also known as SFTS virus (SFTSV), belonging to the Phenuiviridae family and the Bandavirus genus, which was first isolated from patient serum by the Chinese Centre for Disease Control and Prevention in 2010 [1]. The main features of SFTS include fever, thrombocytopenia, leukopenia and gastrointestinal symptoms, and in severe cases, patients may present with multiple‑organ failure (MOF) symptoms such as shock, respiratory failure, disseminated intravascular coagulation (DIC) and death, with a mortality rate of 5–30% in East Asia [2, 3]. SFTS may also present with central nervous system (CNS) involvement, which can severely affect the patient’s disease progression and prognosis and is manifested by seizures, psychiatric symptoms, cognitive impairment, and disorders of consciousness [4, 5]. However, reports of patients who present with CNS symptoms as the first symptom and with marked persistent involuntary shaking of the perioral area and limbs are rare.

## Case presentation

A 69-year-old female patient was admitted to the hospital with fever for 4 days, involuntary shaking around the mouth and limbs for 3 days, and mental abnormalities for 1 day. The patient was admitted to the emergency department of another hospital 4 days before admission because of fever, where her body temperature reached 38.7 °C and she showed poor mental status, less talking, a loss of appetite, but no headache, vomiting, and limb twitching. A routine blood examination showed a white blood cell (WBC) count of 2.28 × 10^9^/L and a platelet count of 165 × 10^9^/L. When given a cooling infusion for symptomatic treatment, her body temperature would temporarily return to normal. Three days before admission, she experienced persistent involuntary trembling around the mouth and lips, as well as trembling of the tongue and extremities. The trembling of the lips, mouth, and both distal upper limbs was especially bothersome and was aggravated by emotional excitement and accompanied by slurred speech. Two days before admission, she had persistent fever, with a body temperature up to 39.6 °C, and the effect of antipyretic drugs was not good. A routine blood examination performed in another hospital showed a WBC count of 1.78 × 10^9^/L and a platelet count of 81 × 10^9^/L, which was significantly decreased compared with the count from the previous examination. One day prior to admission, the patient experienced babbling, restlessness, irritability, and a decline in time and place orientation and calculation power.

The patient had a many-year history of hypertension, diabetes mellitus and hyperlipidaemia; denied a history of working and living in hilly, forested and mountainous areas and travelling; denied a recent history of mosquito bites; and reported a history of close contact with a pet dog in the last month.

Neurological examination after admission showed that the patient had normal arousal but had unclear speech, hyperactivity, irritability. Her time and place orientation and calculation power decreased. The patient was uncooperative in the pharyngeal reflex examination, and involuntary tongue twitching could be seen when the tongue was stretched out. The remaining cranial nerve examination did not show any abnormalities. Perioral and limb involuntary shaking was obvious and persistent, especially in the perioral area and distal part of both upper limbs. Bilateral tendon reflexes were symmetrical, bilateral pathological signs were negative, and meningeal irritation signs were negative.

On admission, viral encephalitis was considered, and intravenous acyclovir antiviral therapy (0.5 g, q8h) was empirically administered. A comprehensive examination revealed that the patient had MOF: (1) Her platelet count further decreased to 63 × 10^9^/L (normal: 100–300 × 10^9^/L), toxic granules were seen in some granulocytes of the peripheral blood smear, and heterogeneous lymphocytes accounted for 21% of the total. (2) She had impaired liver function with elevated liver enzymes (alanine aminotransferase (ALT), 76 IU/L (normal: 5–40 IU/L); aspartate aminotransferase (AST), 188 IU/L (normal: 8–40 IU/L); and gamma-glutamyl transpeptidase (γ-GT), 177 IU/L (normal: 7–50 IU/L)), which was treated with magnesium isoglycyrrhizinate injection and vitamin C for liver protection. (3) She had acute myocardial injury, with an increased heart rate of > 120 beats/minute and markedly elevated myocardial enzyme and B-type natriuretic peptide levels (myoglobin, 299 ng/mL (normal: 25–58 ng/mL); troponin T, 209 ng/L (normal: 0–14 ng/L); and B-type natriuretic peptide, 9,355 pg/mL (normal: 0-125 pg/mL)). Electrocardiograms (ECGs) showed various atypical manifestations, such as short PR intervals; atrial premature, mild ST-segment depression in leads V2V3; and T-wave changes in multiple leads. Cardiac ultrasound showed a normal left ventricular ejection fraction but abnormal segmental motion of the left ventricular wall, biventricular diastolic insufficiency and a small amount of pericardial effusion. Coenzyme Q10 and trimetazidine were given to improve myocardial energy metabolism, and fluid intake and output were closely monitored. (4) The patient had a bacterial infection of the lungs, combined with type I respiratory failure, which were treated with tracheal intubation and mechanical ventilation immediately to assist respiration and antibiotic antimicrobial therapy. The patient did not have prolonged hypoxic injury. (5) She had impaired renal function, with elevated blood urea nitrogen (BUN) (17.33 mmol/L) (normal: 1.7–8.3 mmol/L) and urinary protein. We administered measures to ensure fluid intake and without the use of nephrotoxic drugs. (6) She had impaired pancreatic function, with elevated lipase (56.5 U/L) (normal: 5.6–51.3 U/L); we administered acid-suppressing drugs to inhibit pancreatic secretion and reduce the load and damage to pancreatic tissue. (7) She had abnormal coagulation, with a prolonged prothrombin time (PT) and thrombin time (TT) (15.7 s (normal: 11–15 s) for PT and 22.6 s (normal: 14–21 s) for TT), decreased fibrinogen (1.8 g/L) (normal: 2–4 g/L), and markedly elevated plasma D-dimer (9.01 µg/mL) (normal: 0.01–0.5 µg/mL) and fibrinogen degradation products (FDPs) (28.36 µg/mL) (normal: 0–5 µg/mL). (8) A thrombus had formed in her right peroneal vein and the intermuscular veins of the right and left calves, for which low molecular heparin anticoagulation was given. (9) Her muscle enzyme profiles were variably elevated (creatine kinase (CK), 335 IU/L (normal: 24–195 IU/L); lactate dehydrogenase (LDH), 1347 IU/L (normal: 109–245 IU/L); and alpha-hydroxybutyrate dehydrogenase (α-HBDH), 645 IU/L (normal: 72–182 IU/L)), correlating with inflammatory response-mediated organ damage. (10) The patient experienced a cytokine storm, with significantly increased inflammatory factors (ferritin > 1500 ng/mL (normal: 11-306.8 ng/mL), interleukin (IL)-6 = 49.88 pg/mL (normal: 0–20 pg/mL), IL-8 = 45.99 pg/mL (normal: 0-21.4 pg/mL), and IL-10 = 25.67 pg/mL (normal: 0-5.9 pg/mL), interferon (IFN)-α = 9.76 pg/mL (normal: 0-7.9 pg/mL), and IFN-γ = 18.7 pg/mL (normal: 0-17.3 pg/mL)) in serum (Table 1). (11) Finally, the patient showed an electrolyte balance disorder, as evidenced by hypernatremia (154 mmol/L) (normal: 135–145 mmol/L), hyperchloremia (119 mmol/L) (normal: 96–108 mmol/L), hypocalcaemia (1.92 mmol/L) (normal: 2.03–2.67 mmol/L), and hypophosphatemia (0.54 mmol/L) (normal: 0.84–1.65 mmol/L), and treatments included calcium supplementation, phosphorus supplementation, nasal administration of plain water, and a reduction of sodium and chlorine intake.

**Table 1:** Summary of cytokine levels in CSF and blood

Test items	Results						Reference range
	CSF			Whole blood			
	Day 2 of admission	Day 22 of admission		Day 2 of admission	Day 12 of admission	Day 22 of admission	
IL-2 (pg/mL)	0.27	1.17		0	1.33	1.81	0-11.4
IL-4 (pg/mL)	1.11	0		0.88	1.11	1.54	0-12.9
IL-5 (pg/mL)	0.13	0		0.14	0.13	0	0-3.4
IL-6 (pg/mL)	27.46	4.35		49.88	51.69	12.22	0–20
IL-8 (pg/mL)	546.93	96.17		45.99	15.33	4.62	0-21.4
IL-1β (pg/mL)	1	0		1.11	1.83	0.89	0-12.1
IL-17 A (pg/mL)	0	17.55		3.61	8.01	0	0-20.6
IL-10 (pg/mL)	1.5	0		25.67	1.58	1.27	0-5.9
TNF-α (pg/mL)	1.38	0		1.53	1.09	2.13	0-5.5
IFN-α (pg/mL)	4.47	0.03		9.76	0	0	0-7.9
IL-12P70 (pg/mL)	0	0		0.28	0	0	0-3.2
IFN-γ (pg/mL)	1.14	0.74		18.7	1.98	1.14	0-17.3

*CSF*, cerebrospinal fluid; *IL*, interleukin; *TNF*, tumour necrosis factor; *IFN*, interferon

Lumbar puncture was performed on the second day after admission (Table 2). Cerebrospinal fluid (CSF) was colourless and clear, with a pressure of 190 mmH_2_O (normal: 80–180 mmH_2_O) and a WBC count of 3 × 10^6^/L. CSF cytology showed scattered lymphocytes and a few mononuclear cells. The glucose level and protein counts were normal, chloride was slightly elevated (134 mmol/L) (normal: 118–128 mmol/L), immunoglobulins (Ig) were slightly elevated (IgA, 1.03 mg/dL (normal: 0-0.2 mg/dL); IgM, 0.22 mg/dL (normal: 0-0.2 mg/dL); and IgG, 6.68 mg/dL (normal: 0.48–5.86 mg/dL)), and CSF cytokine levels of IL-6 (27.46 pg/mL) (normal: 0–20 pg/mL) and IL-8 (546.93 pg/mL) (normal: 0-21.4 pg/mL) were elevated. CSF was negative for an autoimmune encephalitis antibody profile (NMDAR, CASPR2, AMPAR1, AMPAR2, LGI1, GABABR, DPPX, and IgLON5), neuroparaneoplastic syndrome antibody profile (Hu, Ri, Yo, CV2, Amphiphysin, GAD65, PNMA2, Recoverin, SOX1, Titin, Tr, and Zic4), and CNS demyelination antibody profile (AQP4, GFAP, MBP, and MOG). Metagenomic next-generation sequencing (mNGS) showed that the number of sequences of a novel Bunyavirus of the Bandavirus genus was 59 in the blood and 12 in the CSF. We also excluded acute febrile illnesses by serum and CSF mNGS, such as dengue fever, chikungunya fever, EB virus infection, renal syndrome hemorrhagic fever, and rickettsial disease.

**Table 2:** Summary of the CSF tests

Test items	Results		Reference range
	Day 2 of admission	Day 22 of admission	
Pressure (mmH_2_O)	190	110	80–180
Total cell count (×10^6^/L)	3	4	0–8
Leukocyte count (×10^6^/L)	3	4	0–8
Protein (mg/dL)	39.3	27.2	15–45
Glucose (mg/dL)	76.68	93.42	45–80
Chloride (mmol/L)	134	130	118–128
IgA (mg/dL)	1.03	0.33	0-0.2
IgM (mg/dL)	0.22	0.06	0-0.2
IgG (mg/dL)	6.68	4.3	0.48–5.86
Antibodies against NMDAR, CASPR2, AMPAR1, AMPAR2, LGI1, GABABR, DPPX and IgLON5	negative	—	negative
Antibodies against Hu, Ri, Yo, CV2, Amphiphysin, GAD65, PNMA2, Recoverin, SOX1, Titin, Tr and Zic4	negative	—	negative
Antibodies against AQP4, GFAP, MBP, and MOG	negative	—	negative
Specific IgG oligoclonal bands	negative	negative	negative
Band pattern	type I polyclonal band	type I polyclonal band	type I polyclonal band
Metagenomic next-generation sequencing	positive	negative	negative

*CSF*, cerebrospinal fluid; *Ig*, immunoglobulin; *NMDAR*, N-methyl-D-aspartate receptor; *CASPR2*, contactin associated protein 2; *AMPAR*, α-amino-3-hydroxy-5-methyl-4-isoxazolepropionic acid receptor; *LGI1*, leucine-rich glioma inactivated 1; *GABABR*, gamma aminobutyric acid receptor type b; *DPPX*, dipeptidyl-peptidase-like protein-6; *IgLON5*, immunoglobulin-like cell adhesion molecule 5; *GAD65*, glutamic acid decarboxylase 65; *PNMA2*, paraneoplastic antigen MA2; *SOX1*, sex-determining region of Y chromosome-related high mobility group box 1; *AQP4*, aquaporin protein-4; *GFAP*, glial fibrillary acidic protein; *MBP*, myelin basic protein; *MOG*, myelin oligodendrocyte glycoprotein

A diagnosis of SFTS that started with symptoms of CNS and encephalitis due to a novel Bunyavirus was considered based on the patient’s clinical presentation and laboratory test results. With immediate effect, acyclovir was adjusted to the broad-spectrum antiviral drug Foscarnet sodium (3 g, q8h); intravenous infusion of dexamethasone (10 mg qd for five days) and intravenous immunoglobulin (IVIG) (0.4 g/kg for five days) were administered to regulate immune function and inhibit the cytokine storm; nifedipine and benidipine hydrochloride were given to reduce the viral-induced calcium inflow to inhibit viral replication, reduce the viral load and increase the platelet count; clonazepam (1 mg, q8h) was given to relieve the patient’s obvious symptoms of involuntary shaking; and adequate symptomatic supportive therapy was given to ensure adequate calorie and protein intake and to maintain water, electrolyte, blood glucose and acid‒base balance.

**Fig. 1:**
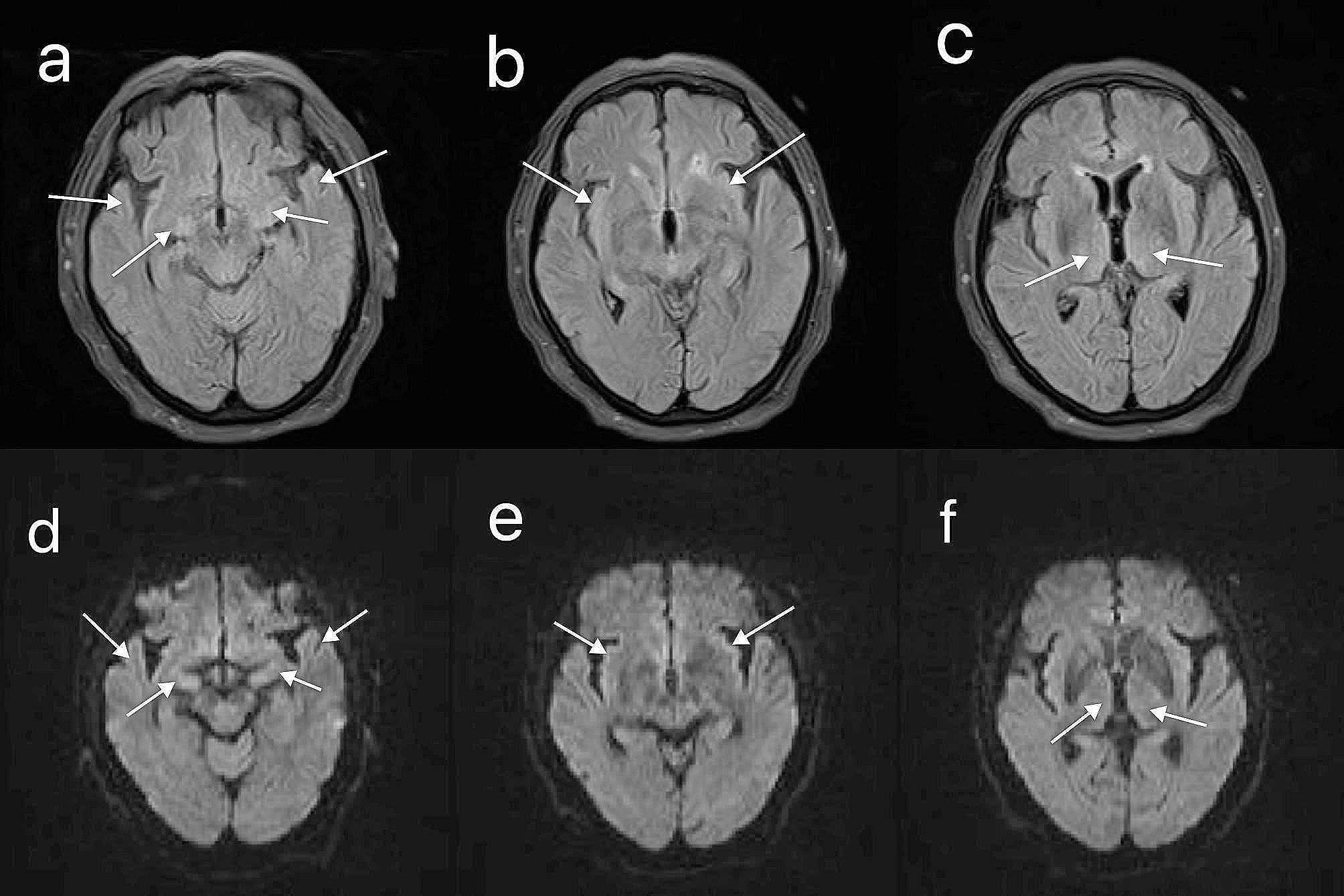
Cranial MRI of the patient 12 days after admission. Bilateral anterior temporal lobe **(a and d)**, temporal lobe leptomeningeal gyrus **(a and d)**, insular cortex **(b and e)**, and bilateral thalamus **(c and f)** FLAIR and DWI sequences with slightly high signals

**Fig. 2:**
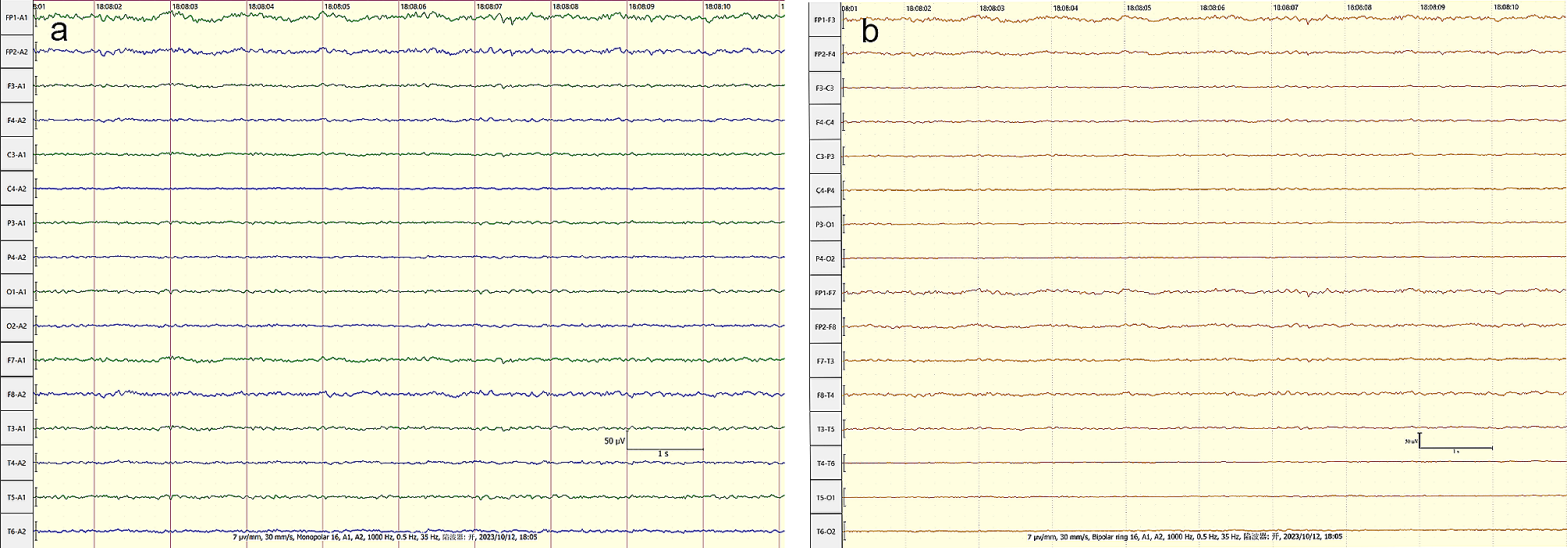
Sixteen-lead resting-state EEG of the patient 16 days after admission. Simultaneous display an EEG record in monopolar and bipolar montages. A low-amplitude state can be seen in all leads. **(a)** monopolar montage EEG, **(b)** bipolar montage EEG

After 3 days of hospitalization, the patient’s platelet and WBC counts began to rise gradually and returned to normal levels. After 5 days of hospitalization, the patient’s involuntary shaking and psychiatric symptoms were less severe than before, but compliance with activities was still poor, and her cognitive level still had not returned to normal. After 11 days of hospitalization, the lung infection was better than before, and ventilator withdrawal training was started. After 12 days of hospitalization, cranial magnetic resonance imaging (MRI) was performed, which showed slightly high signals in the bilateral anterior temporal lobe, temporal lobe hook gyrus, insular cortex, and bilateral thalamus on fluid attenuated inversion recovery (FLAIR) and diffusion weighted imaging (DWI) (Fig. 1a-f). After 13 days of hospitalization, a blood sample was negative for novel Bunyavirus nucleic acid. After 16 days of hospitalization, her condition was significantly better than before, she could perform activities as instructed and answer questions correctly, her time and place orientation returned to normal, and her cognitive level was better than before. A electroencephalogram (EEG) was performed, and a full-lead low-wave amplitude state was observed (Fig. 2). After 17 days of hospitalization, the ventilator was completely withdrawn, and the tracheal tube was removed. A repeat lumbar puncture 3 weeks after hospitalization showed a pressure of 110 mmH_2_O, a WBC count of 4 × 10^6^/L, a normal protein count, a slightly elevated glucose level (5.19 mmol/L, compared with a glucose of 7.9 mmol/L over the same period), a slightly elevated chlorine level (130 mmol/L), and a return of Ig to normal. The levels of cytokines IL-6 (4.35 pg/mL) and IL-8 (96.17 pg/mL) decreased significantly compared with the previous levels, and the levels of whole-blood cytokines returned to the normal range (IL-6, 12.22 pg/mL; IL-8, 4.62 pg/mL; IL-10, 1.27 pg/mL; IFN-α, 0 pg/mL; and IFN-γ, 1.14 pg/mL) in serum (Table 1). No further novel Bunyaviruses were detected by mNGS of the CSF. Meanwhile, MOF gradually recovered, and liver, heart, lung, kidney, pancreas and coagulation function; the muscle enzyme profile; inflammatory factors; and electrolyte levels gradually returned to normal levels.

After antiviral therapy, immunotherapy, life support and symptomatic treatment, the patient’s vital signs were stable 3 weeks after admission, with clear speech and normal higher cortical function to perform tasks correctly on command. The muscle strength of all four limbs was grade 5, muscle tone was normal, bilateral tendon reflexes existed symmetrically, an ataxia test was normal, bilateral pathological signs were negative, and meningeal irritation signs were negative. She was discharged from the hospital in 23 days after admission. The patient was followed up 1 month after she was discharged from the hospital and is now back to her normal living conditions, with normal functioning of the higher cortex, the ability to take care of herself, and the ability to perform all of the activities she regularly engages in.

## Discussion and conclusions

SFTS is an infectious disease caused by SFTSV infection. The epidemic period is mainly in May-August, and SFTSV is mainly transmitted by tick bites to humans. In recent years, interpersonal and human-animal transmission has also been found. An epidemiological survey of SFTS found that 48% of the patients had had close contact with their pets within two weeks of the onset of the disease [6]. The general population is susceptible, with a higher risk of infection in residents living in areas such as hills, mountains and forests and in people who spend time outdoors. In this case, SFTSV was isolated from blood and CSF. There was no history of tick bites or travel in the wild, but there was a history of close contact with a pet dog within the past month, and we hypothesized that the infected dog might have been the source of SFTSV in this patient.

The pathogenesis of CNS involvement in SFTS patients is unclear. Previous studies have demonstrated that Bunyaviruses have neurological properties of attack, and Park et al. found viral transcripts of novel Bunyaviruses in the brain and spinal cord of an aged model ferret. It is hypothesized that novel Bunyaviruses also involve the CNS, with consequent symptoms [7]. Possible mechanisms by which SFTSV attacks the CNS include direct invasion, cytokine storms, and impaired immune function. Kaneko et al. [8] performed an autopsy on a patient with SFTS with rapid CNS involvement, and the pathological findings revealed a massive infiltration of macrophages with high haematoxylin content and inflammatory cells around the microvessels of the cerebral pontine, fibrin deposition in the vessels, and focal degenerative lesions in some neuronal cells. In a variety of brain tissues, positive SFTSV nucleocapsid protein antigens were observed in the immunoblasts infiltrating the vascular lumen, suggesting that SFTSV can invade the CNS directly for disease development. The availability of agents that recognize these antigens also suggest immunoassays are possible and available for serodiagnosis. For example, serum enzyme linked immunosorbent assay or immunofluorescence to determine SFTSV antigens and antibodies have been used for clinical diagnosis [9]. Several studies [10–15] have found that the blood levels of several cytokines, including IFN-α, IFN-γ, IL-6, IL-8, IL-10, tumour necrosis factor (TNF)-α, and monocyte chemotactic protein (MCP)-1, are elevated in patients with SFTS, and IL-8 and MCP-1 levels in the CSF are significantly higher than the blood of those who present with CNS symptoms [10], suggesting that a cytokine storm may increase vascular permeability and prompt SFTSV to cross the blood‒brain barrier (BBB) and invade the CNS. SFTSV was found in the CSF of this patient, suggesting that the virus had invaded the patient’s CNS. The patient’s blood levels of IL-6, IL-8, IL-10, IFN-α, and IFN-γ were markedly elevated compared with normal ranges; IL-6 and IL-8 were elevated in the CSF; and CSF IL-8 levels were significantly higher than the blood levels, which was consistent with the results of a previous study [10–15], further suggesting that the cytokine storms induced by multiple elevated cytokines may increase BBB vascular permeability and contribute to the SFTSV invasion of the CNS. In patients with SFTS complicated by neurological involvement, protein and glucose levels in the CSF are normal and that an increase in leukocytes in the CSF may be uncommon. However, in the case of a high suspicion both on a clinical and epidemiological level in countries where the infection exists, in these patients the search for MCP-1 and IL-8 in the CSF and serum is indicated and CSF viral RNA detection are recommended.

According to the course of infection, SFTS can be divided into four periods: the incubation period, the febrile period, the MOF period, and the recovery period [1–3, 16]. Patients with SFTS can present with neurological symptoms, which usually appear approximately 5 days after the onset of the disease (Table 3) and are often regarded as a complication of SFTS, which has been referred to as SFTS-associated encephalopathy/encephalitis (SFTSAE) [10]. SFTSAE mainly manifests as headache, seizures, mental abnormality, irritability, limb convulsions, cognitive impairment, and impaired consciousness, with an incidence of approximately 19.1-57.02% [4, 5, 11, 17]. Most patients with SFTSAE develop impaired consciousness, such as coma, before their condition is taken seriously, which leads to a poor prognosis for the patients [4, 18]. Most clinicians rely on the clinical manifestations to make the clinical diagnosis. SFTSV has rarely been isolated from CSF. We screened studies and case reports of SFTS with CNS involvement and found no reports of disease onset with CNS symptoms such as marked persistent involuntary shaking of the perioral area and extremities. In this case, the patient first presented with fever, followed by persistent involuntary tremors of the perioral area and limbs and mental behavioural abnormalities such as rambling, irritability and agitation; furthermore, the whole-genome sequence of SFTSV was found by mNGS of blood and CSF. The case reported here is a case of SFTS with CNS symptoms onset, accompanied by perioral and extremity persistent involuntary shaking, which has not been previously reported in the literature. It has been reported in the literature that SFTS patients can have tremors of limbs and muscles [8, 17, 19], but most of them occurred in the middle and late stages of the disease, and the tremor amplitude was small. In this case, the patient had large-amplitude involuntary shaking of the limbs that was persistent and intensified during agitation, which immediately attracted the clinician’s attention. An additional movie file shows this in more detail [see Additional file 1]. However, the specific underlying mechanism is not clear, and a description of similar symptoms of viral encephalitis and an analysis of the underlying mechanism have not been found before; therefore, further studies are needed. The course of the disease in this patient was consistent with the general pattern, with the clinical experience of the febrile period, the MOF period, and the recovery period. The febrile period lasted approximately 4 days, followed by MOF involving the liver, heart, lungs, kidneys, and pancreas, and then the recovery period began approximately 2 weeks after the disease onset, with clinical symptoms gradually returning to normal.

There are fewer reports on neurological-related ancillary investigations (CSF, cranial imaging, and EEG) in SFTS patients with CNS involvement, and we analyse this because SFTS patients rarely start with CNS symptoms and go directly to the neurology department and because such patients are generally more severely ill, making it difficult for them to cooperate in completing the relevant investigations. In a few previous studies, lumbar puncture CSF tests in SFTS patients with CNS symptoms were mostly normal, with few abnormal changes in leukocyte counts, sugars and proteins [10, 20]. Park et al. [10] analysed head imaging and EEG in a series of SFTS patients presenting with CNS symptoms, and no new focal lesions were seen on imaging in any of the brain parenchyma, suggesting that the imaging was not specific and that the EEG in the majority of the patients showed a slow-wave background rhythm (δ-θ), a common feature of encephalitis/encephalopathy. In this patient, two lumbar punctures were performed successively, and no CSF leukocyte abnormalities were observed in any of them either; it was presumed that SFTSV infection was less likely to involve the meninges. We performed cranial MRI and EEG on the patient 12 and 16 days after admission, respectively, and slightly high signals were observed in the bilateral anterior temporal lobes, temporal lobe hook gyrus, insular cortex, and bilateral thalamus in the FLAIR and DWI sequences of cranial MRI, all of which were consistent with the general imaging manifestations of viral encephalitis and were presumed to be related to viral invasion. In addition, we should consider the similarities and differences between the above MRI changes and cortical laminar necrosis associated with hypoxia or hypotension. We found that both had MRI high signals distributed along the cortex. However, this patient’s cranial MRI showed cortical high signals only in FLAIR and DWI sequences, and no abnormal signal was found in T1WI, which was the most obvious difference from cortical laminar necrosis. Furthermore, the patient did not show hypotension or significant hypoxic injury, so the changes on cranial MRI were more likely to be inflammatory changes of viral encephalitis and less relevant to cortical laminar necrosis. The background rhythm of the EEG was an α rhythm, and the whole leads were in low amplitude, which was different from previous studies [10]. It was presumed that the patient’s brain inflammation had tended to recover at that time, but the suppression of cortical function was remained.

There are no specific drugs for the treatment of CNS symptoms in SFTS, and symptomatic supportive treatment is the mainstay. In vitro and ex vivo studies have found that nifedipine or benidipine hydrochloride can inhibit SFTSV replication, reduce viral load, increase platelet counts, and reduce morbidity and mortality, as confirmed in a retrospective clinical study [21, 22]. Glucocorticoids can inhibit the cytokine storms caused by the overproduction of cytokines and reduce patient mortality [12, 13, 23], and a Japanese report documented that three SFTS patients with impaired consciousness recovered without any neurological sequelae after short-term glucocorticoid treatment. However, the authors also suggested that the dosage should be minimized and the duration of administration should be shortened to inhibit cytokine storms and provide systemic benefit, rather than high doses or prolonged use, to avoid side effects [24]. Gamma globulin, which triggers complement activation and viral neutralization and influences the differentiation process of Schwann cells to increase their regenerative potential [25], has been used to treat other virus-induced encephalitides and can be used for the treatment of CNS symptoms in SFTS. Two successful cases of combined glucocorticoid and IVIG therapy were reported in Korea [26]. Two case reports documented that plasma exchange therapy reduced cytokine levels but not viral load, presumably making plasma exchange more effective at an early stage [27, 28]. However, these are case reports, and the findings should be confirmed by large-scale randomized controlled studies. In this case, the patient was given the broad-spectrum antiviral drug foscarnet sodium, intravenous infusion of dexamethasone and IVIG to regulate the immune function of the body and inhibit the inflammatory storm, nifedipine and benidipine hydrochloride to inhibit viral replication and reduce the viral load, and other symptomatic treatments. The patient’s clinical manifestations and laboratory indicators gradually improved.

The prognosis of patients with SFTS is related to numerous factors, and studies have shown that advanced age; significant elevations in ALT, AST, CK, CK-MB, LDH, γ-GT, and BUN; low platelet count; persistent lowering of blood calcium; and the presence of CNS symptoms are all important influences that can lead to a poor prognosis [29–34]. Most of these are commonly used to monitor cardiac, hepatic and renal function, and significant abnormalities in their results indicate more severe organ damage and dysfunction. In addition, there is a statistically significant difference in serum viral copy number between deceased and non-dead patients. The mean viral copy number was higher in deceased patients than in surviving patients, and patients with higher copy numbers had higher mortality rates [35, 36]. It was shown that the serum viral load detected by polymerase chain reaction (PCR) on admission was higher in SFTSAE patients than in non-encephalitis patients [11]. The above suggests a relationship between patient serum number of SFTSV RNA copies and encephalitis CNS symptoms and mortality in SFTS patients. CNS symptoms are often considered to be associated with fatal outcomes in patients with SFTS [33], and early diagnosis and treatment of neurological symptoms can help reduce mortality. Advanced age; long intervals between onset and admission; comorbid diabetes mellitus or subcutaneous haemorrhage; pulmonary rales; low platelet count; elevated neutrophil percentages and LDH, CK, and C-reactive protein (CRP) levels; and decreased chloride concentrations are significantly associated with the development of CNS symptoms and should be taken into consideration in clinical practice [11, 17]. We believe that changes in platelet count and CK-MB should be monitored in patients with SFTSAE. As shown in previous, decreased platelet counts and high CK-MB levels are risk factors for poor prognosis in patients with SFTS. The presence of encephalitis is evidence of a more critical condition. Monitoring changes in platelet counts may provide an initial indication of the direction of the patient’s regression. It has been found that in cardiac enzyme profiles, patients presenting with CNS symptoms have elevated CK levels earlier than LDH and AST levels, and elevated liver enzyme levels later than cardiac enzymes [17]. Therefore, early monitoring of CK-MB levels may have a predictive effect on the development of CNS symptoms in patients. Although the mortality rate of SFTS patients presenting with CNS symptoms is significantly higher [11], several studies have found [11, 37, 38] that the long-term prognosis of surviving patients is good, with no obvious sequelae after active treatment. In this case, the patient’s laboratory indicators were consistent with the factors leading to a poor prognosis, and the CNS symptoms were prominent, suggesting that the condition was critical, but with timely administration of treatment, the patient’s condition eventually returned to normal.

In summary, we report a case of SFTS in a patient who started with CNS symptoms accompanied by marked persistent involuntary perioral and extremity shaking, and the whole-genome sequence of SFTSV was found by mNGS of both serum and CSF (It is important to note that hospitals where mNGS analysis is unavailable should use real-time fluorescent quantitative PCR to detect SFTS-specific nucleic acids in serum and CSF.). This has given us great insight into the fact that SFTS should be considered a possible cause when patients present with common CNS symptoms of viral encephalitis, such as mental behavioural abnormalities, convulsions, and cognitive deficits, or rare symptoms, such as persistent involuntary shaking of the perioral area and limbs in the rare case of this patient, combined with thrombocytopenia and leukopenia. Prompt lumbar puncture examination for SFTSV should be performed, and appropriate treatment should be given aggressively to reduce mortality.

**Table 3:** CNS involvement in relation to the timing of the clinical onset of SFTS

Author(s)/year	Clinical manifestations of CNS symptoms	Time after the onset of illness
Cui et al.,2015 [11]	Headache, vomiting, disorders of consciousness	A median of 5 days (range, 2–14 days)
Park et al.,2016 [28]	Confused verbal responses, lacked orientation of time and place, coma	8 days
Kawaguchi et al.,2016 [38]	Disturbance of consciousness, myoclonus to the face and limbs	7 days
Nakamura et al.,2017 [24]	Disturbance of consciousness	3 days/6 days/11 days
Kaneko et al.,2017 [8]	Limb tremor, slurred speech, disturbance of consciousness	9 days
Kim et al.,2018 [39]	Stuporous mentality	6 days
Park et al.,2018 [10]	Headache, altered mental status, disorientation, tremor, seizure, dysarthria	3–7 days
Sun et al.,2019 [40]	Disturbance of consciousness, convulsions	5 days
Wang et al.,2020 [41]	Memory impairment, epileptic episodes	4 days
Li et al.,2021 [16]	Mental status, consciousness disorder, seizure	Approximately 5 days
Casel et al.,2021 [4]	Lethargy, muscular tremors, convulsions, coma	Frequently occur in the terminal stage
Fei et al.,2023 [5]	Tremor, cognitive dysfunction, consciousness disorder, convulsions, seizures	Survivors group:7.47 ± 1.91 days/Non-survivors group:7.2 ± 3.16 days
Xu et al.,2023 [37]	Consciousness disorder, headache, restlessness, tremor, convulsion, mental state change	The median time was 5 days

*CNS*, central nervous system; *SFTS*, severe fever with thrombocytopenia syndrome

### Electronic supplementary material

Below is the link to the electronic supplementary material.

Supplementary Material 1. File name: Additional file 1. File format: mp4. Title of data: Video of patient with persistent involuntary shaking of the perioral area and limbs. Description of data: We took this video on day 2 after the patient was admitted to the hospital. The patient develops persistent involuntary shaking of the perioral area and limbs, especially in the perioral area and distal limbs, which is aggravated by agitation and is accompanied by slurred speech.

Supplementary Material 2. CARE Checklist of information to include when writing this case report.

## Data Availability

No datasets were generated or analysed during the current study.

## Abbreviations

SFTS: severe fever with thrombocytopenia syndrome
SFTSV: severe fever with thrombocytopenia syndrome virus
MOF: multiple-organ failure
DIC: disseminated intravascular coagulation
CNS: central nervous system
mNGS: metagenomic next-generation sequencing
CSF: cerebrospinal fluid
ALT: alanine aminotransferase
AST: aspartate aminotransferase
γ-GT: gamma-glutamyl transpeptidase
ECG: electrocardiogram
BUN: blood urea nitrogen
PT: prothrombin time
TT: thrombin time
FDPs: fibrinogen degradation products
CK: creatine kinase
LDH: lactate dehydrogenase
α-HBDH: alpha-hydroxybutyrate dehydrogenase
IL: interleukin
IFN: interferon
Ig: immunoglobulin
IVIG: intravenous immunoglobulin
MRI: magnetic resonance imaging
FLAIR: fluid attenuated inversion recovery
DWI: diffusion weighted imaging
EEG: electroencephalogram
TNF: tumour necrosis factor
MCP: monocyte chemotactic protein
BBB: blood-brain barrier
SFTSAE: severe fever with thrombocytopenia syndrome -associated encephalopathy/ encephalitis
PCR: polymerase chain reaction
CRP: C-reactive protein
