# Supplementary material for: Severe fever with thrombocytopenia syndrome with central nervous system symptom onset: a case report and literature review
Source: BMC Neurol. 2024 May 10;24:158. doi: 10.1186/s12883-024-03664-6 (PMC11084135; doi:10.1186/s12883-024-03664-6)
Supplement: Supplementary file 2 — Supplementary Material 2. CARE Checklist of information to include when writing this case report. [file 12883_2024_3664_MOESM2_ESM.pdf]

CARE case report guidelines

CARE Checklist of information to include when writing a case report

(cc) BY-NC-ND

✓

Topic	Item	Checklist item description	Reported on Line
Title	1	The diagnosis or intervention of primary focus followed by the words "case report"	1. 2
Key Words	2	2 to 5 key words that identify diagnoses or interventions in this case report, including "case report"	25-26
Abstract (no references)	3a	Introduction: What is unique about this case and what does it add to the scientific literature?	12-14
	3b	Main symptoms and/or important clinical findings	15-16
	3c	The main diagnoses, therapeutic interventions, and outcomes	16-17 19-20
	3d	Conclusion—What is the main "take-away" lesson(s) from this case?	21-24
Introduction	4	One or two paragraphs summarizing why this case is unique (**may include references**)	28-39
Patient Information	5a	De-identified patient specific information.	41
	5b	Primary concerns and symptoms of the patient.	42-56
	5c	Medical, family, and psycho-social history including relevant genetic information	57-60
	5d	Relevant past interventions with outcomes	57
Clinical Findings	6	Describe significant physical examination (PE) and important clinical findings.	61-68
Timeline	7	Historical and current information from this episode of care organized as a timeline	161. 162. 166
Diagnostic Assessment	8a	Diagnostic testing (such as PE, laboratory testing, imaging, surveys).	69-70 106-117
	8b	Diagnostic challenges (such as access to testing, financial, or cultural)	—
	8c	Diagnosis (including other diagnoses considered)	69-70
	8d	Prognosis (such as staging in oncology) where applicable	—
Therapeutic Intervention	9a	Types of therapeutic intervention (such as pharmacologic, surgical, preventive, self-care)	118-127
	9b	Administration of therapeutic intervention (such as dosage, strength, duration)	118-127
	9c	Changes in therapeutic intervention (with rationale)	128-151
Follow-up and Outcomes	10a	Clinician and patient-assessed outcomes (if available)	152-157
	10b	Important follow-up diagnostic and other test results	157-160
	10c	Intervention adherence and tolerability (How was this assessed?)	—
	10d	Adverse and unanticipated events	—
Discussion	11a	A scientific discussion of the strengths AND limitations associated with this case report	—
	11b	Discussion of the relevant medical literature **with references**.	170-259
	11c	The scientific rationale for any conclusions (including assessment of possible causes)	203-231
	11d	The primary "take-away" lessons of this case report (without references) in a one paragraph conclusion	291-299
Patient Perspective	12	The patient should share their perspective in one to two paragraphs on the treatment(s) they received	—
Informed Consent	13	Did the patient give informed consent? Please provide if requested	Yes ☑ No ☐
